# Lessons learnt from the first efficacy trial of a new infant tuberculosis vaccine since BCG

**DOI:** 10.1016/j.tube.2013.01.003

**Published:** 2013-03

**Authors:** Michele Tameris, Helen McShane, J. Bruce McClain, Bernard Landry, Stephen Lockhart, Angelique K.K. Luabeya, Hennie Geldenhuys, Jacqui Shea, Gregory Hussey, Linda van der Merwe, Marwou de Kock, Thomas Scriba, Robert Walker, Willem Hanekom, Mark Hatherill, Hassan Mahomed

**Affiliations:** a South African TB Vaccine Initiative, Institute of Infectious Disease and Molecular Medicine (IIDMM) and School of Child and Adolescent Health, University of Cape Town, Brewelskloof Hospital, Haarlem Street, Worcester, Western Cape 6850, South Africa; b Jenner Institute, University of Oxford, United Kingdom; c Aeras, Rockville, USA; d Oxford Emergent Tuberculosis Consortium, United Kingdom; e Vaccines for Africa, Institute of Infectious Disease and Molecular Medicine and Department of Medical Microbiology, University of Cape Town, South Africa

**Keywords:** BCG, Vaccine, Tuberculosis, Lessons learnt, Implementation

## Abstract

**Background:**

New tuberculosis (TB) vaccines are being developed to combat the global epidemic. A phase IIb trial of a candidate vaccine, MVA85A, was conducted in a high burden setting in South Africa to evaluate proof-of-concept efficacy for prevention of TB in infants.

**Objective:**

To describe the study design and implementation lessons from an infant TB vaccine efficacy trial.

**Methods:**

This was a randomised, controlled, double-blind clinical trial comparing the safety and efficacy of MVA85A to Candin control administered to 4–6-month-old, BCG-vaccinated, HIV-negative infants at a rural site in South Africa. Infants were followed up for 15–39 months for incident TB disease based on pre-specified endpoints.

**Results:**

2797 infants were enrolled over 22 months. Factors adversely affecting recruitment and the solutions that were implemented are discussed. Slow case accrual led to six months extension of trial follow up.

**Conclusion:**

The clinical, regulatory and research environment for modern efficacy trials of new TB vaccines are substantially different to that when BCG vaccine was first evaluated in infants. Future infant TB vaccine trials will need to allocate sufficient resources and optimise operational efficiency. A stringent TB case definition is necessary to maximize specificity, and TB case accrual must be monitored closely.

## 1 Background

Nine million new cases of tuberculosis (TB) occur worldwide each year and it is estimated that 1 million (11%) are in children under 15 years.^1^ Universal infant vaccination with *Mycobacterium bovis* bacille Calmette-Guérin (BCG), the only licensed TB vaccine, is routine in TB endemic settings.^2^ BCG vaccination coverage is greater than 95% in South Africa^3^ where TB incidence is 981/100,000.^4^ BCG vaccination confers consistent, but partial protection against disseminated forms of TB^2,5^ but protection against pulmonary disease in children and adults is highly variable.^6^ A more effective TB vaccine is needed urgently.

Over ten candidate TB vaccines designed either to boost BCG or replace it are at different stages of clinical testing.^7^ One of these novel vaccines, MVA85A, is a recombinant strain of Modified *Vaccinia virus Ankara* expressing the *Mycobacterium tuberculosis* (*Mtb*) antigen 85A (Ag85A)^8^ and is designed to boost the immune response to BCG. The safety and immunogenicity of MVA85A have been extensively tested in clinical trials in adults, adolescents, children, and infants.^9–19^

The TB vaccine trial landscape has changed markedly in the half century since the infant BCG efficacy trials of Rosenthal and Aronson.^20,21^ Modern clinical trials are conducted in a highly regulated research environment, in which the safety of participants is paramount, guided by the principles of International Conference on Harmonisation Good Clinical Practice (ICH-GCP). The introduction of effective anti-tuberculous chemotherapy for TB disease and isoniazid preventive therapy (IPT) for TB-exposed children, coupled with the need for active surveillance for early detection and treatment, has dramatically shifted the TB phenotype observed in modern vaccine trials towards an early, mild presentation.^22–24^ These considerations impact negatively on the diagnosis of childhood TB as the efficacy endpoint of such trials, even in high TB burden countries, which have traditionally reported cases of advanced childhood TB disease.^23,25^ Further, vaccine-induced protection by any new TB vaccine must be demonstrated *in addition* to that conferred by BCG. It follows that case-finding strategies and definition of TB disease endpoints will be crucial to the successful demonstration of efficacy of any new infant TB vaccine.

A phase IIb proof-of-concept safety and efficacy trial of an infant BCG – prime and MVA85A – boost regimen began enrolment in July 2009, at the field site of the South African Tuberculosis Vaccine Initiative (SATVI) near Cape Town. This is the first infant efficacy trial of a new TB vaccine to be conducted since BCG. We report on the operational and scientific challenges experienced by the study team during the conduct of this trial.

## 2 Methods

### 2.1 Study design

This was a parallel randomized, controlled, double-blind clinical trial with a 1:1 ratio of intervention to control.

### 2.2 Study population

The SATVI field site is set in a region where the TB incidence across all ages is 1400/100,000 and the incidence in children younger than two years of age is estimated at 1500/100,000.^26–28^ The study area has a total population of 350,000 with an annual birth cohort of 7000. Healthy BCG-vaccinated infants aged 4–6 months, without evidence of individual or maternal HIV infection, without evidence of TB exposure or infection, and whose routine immunizations were up to date, were enrolled.

### 2.3 Recruitment, consenting and screening

Mothers of infants of 6 weeks or older were approached for possible participation in the trial. Once informed consent had been signed by the legal guardian, the infants were randomised sequentially into one of five cohorts starting with an initial safety cohort of at least 330 participants, followed by three immunogenicity cohorts of up to 60 participants each and the final, largest correlates of protection cohort of 2400.

### 2.4 Inclusion/Exclusion criteria at commencement of trial

Infants were required to be between 126 and 154 days old at randomisation, to have received BCG vaccination within 7 days of birth, to have received routine doses of EPI vaccines, including pneumococcal vaccine more than 28 days prior to randomisation, and have a weight >10th percentile at randomisation. Infants were excluded if they had an acute illness or fever ≥37.5 °C on Day 0, evidence of infant or maternal HIV infection, evidence of chronic hepatitis, a positive QuantiFERON TB Gold (in-tube) test (QFT) (Cellestis, Victoria, Australia) or a history of known TB disease, treatment of TB, or household TB contact.

### 2.5 Randomization and blinding

Infants confirmed eligible were randomized to receive 0.06 mL of either MVA85A vaccine candidate (1 × 10^8^ plaque forming units [pfu]) or *Candida* skin test antigen control, Candin®, by intradermal injection into the left deltoid region using a masked labeled syringe. Candin was selected for its intradermal administration with similar local reactogenicity to MVA85A, thus ensuring unbiased safety and efficacy assessments. The study pharmacist was the only member of the field team unblinded to allocation of the investigational product. The infants' caregivers and study staff involved in all other aspects of the trial remained blinded to treatment allocation throughout the trial.

### 2.6 Outcomes

The primary objective of this study was to evaluate the safety profile of MVA85A in BCG-vaccinated, HIV-negative infants. The secondary objectives were: 1) to evaluate the efficacy of the MVA85A vaccine compared to placebo control in the prevention of TB disease using three clinical endpoints, 2) the evaluation of the immunogenicity of MVA85A compared to controls and 3) the evaluation of the rate of *Mtb* infection, as defined by QFT conversion at final study assessment in MVA85A recipients compared to controls in infants without a diagnosis of TB disease during the trial.

### 2.7 Follow up

All enrolled infants were followed for efficacy. Infants were to be followed up for between 9 months and 33 months (mean 18 months) by visits to the trial clinic and home visits. Legal guardians were interviewed about the health status of the participant with a focus on possible TB symptoms, and phlebotomy was done per protocol for safety and immunogenicity assessments. Blood was drawn for QFT at Day 336 and at the end of study visit.

### 2.8 Safety endpoints

Adverse events, both local and systemic, were recorded for the first twenty-eight days post-vaccination, and classified for severity and relationship to vaccine. Serious adverse events (SAEs) were collected for the duration of study participation through active surveillance and self-reporting at routine visits. SAEs were reported within specified time frames to the sponsor, regulatory authorities and the ethics committee. An unblinded safety review by the Safety and Monitoring Committee occurred after completion of Day 28 for the initial safety cohort and again after Day 84 for the 1000th participant.

### 2.9 Case accrual and efficacy endpoints

Follow-up visits and active surveillance of hospital records, clinic TB registers, radiology records and death certificates were used to identify suspected cases of TB. A household TB contact would also trigger a case-finding process.^28^ Any infant suspected of TB, or with a history of TB contact, or known tuberculin skin test (TST) or QFT conversion, was admitted to a case verification ward (CV ward) for standardized investigations.^26,28^ Children who were diagnosed as having TB and started on treatment by the health services were where possible admitted to the CV ward as soon as possible after diagnosis for these investigations. A trial-independent, hospital-based TB medical officer reviewed each case at discharge and prescribed appropriate care and treatment. Digital chest X-ray images were reviewed by an independent, blinded panel of three expert paediatric radiologists. A majority agreement on the presence of pre-specified radiological pathology was required for a chest X-ray to be classified as positive for inclusion in the primary efficacy endpoint. The endpoint definitions used to define a TB case are set out in Table 1.

### 2.10 Sample size determination

Given estimated tuberculosis cumulative incidence of 3% over 18 months in the control group, 1392 subjects per study group would provide a 90% chance of detecting a 60% reduction between the treated and control groups based on a two-sided log rank test at a significance level of 0.05. The enrolment target for the study was thus 2784 infants. A sample size of 1392 MVA85A vaccinated subjects would give a greater than 75% chance of observing an adverse event that has an approximately 1 in a 1000 actual rate of occurrence.

### 2.11 Study approvals

The trial was approved by the University of Cape Town (UCT) Faculty of Health Sciences Human Research Ethics Committee (REC), Oxford University Tropical Research Ethics Committee and the South African Medicines Control Council (MCC). Permission was obtained from the Western Cape Department of Health to access their health facilities.

### 2.12 Clinical trials registration

The trial was registered on South African National Clinical Trials Register in November 2008 (DOH-27-0109-2654) and on clinicaltrials.gov in July 2009 (NCT00953927).

## 3 Results

**Figure 1:**
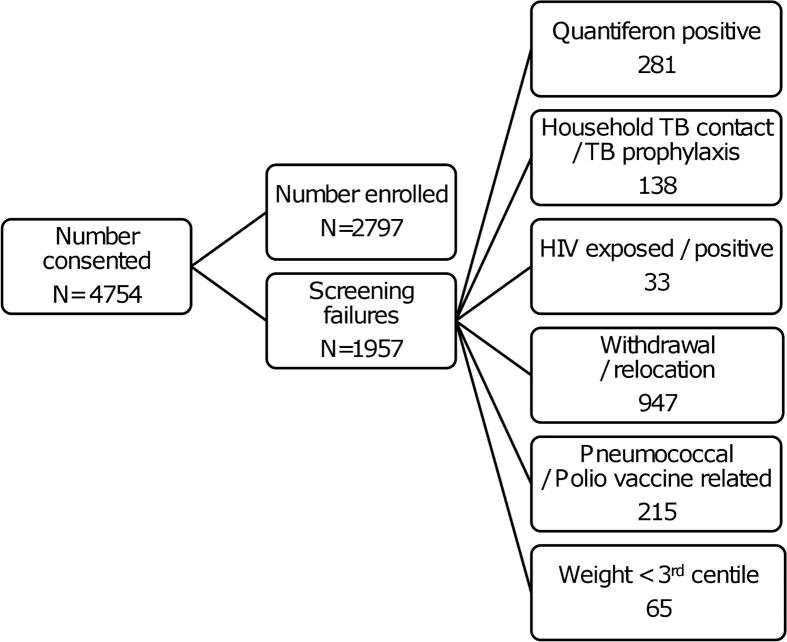
Main reasons for screening failure.

Enrolment commenced in July 2009 and was completed in May 2011. 4754 parents/legal guardians signed consent and 2797 infants were successfully screened and enrolled. The main reasons for exclusion are set out in Figure 1.Slightly more infants were enrolled than targeted because all infants that had already entered screening were allowed to be enrolled although the enrolment target of 2784 had been reached. Closeout visits were completed in September 2012, but evaluation of TB suspects detected through closeout visits continued until October 2012.

Challenges arising from study design elements, operational lessons from implementation of the protocol, and alternative approaches and solutions are summarised in Table 2.

### 3.1 Recruitment

#### 3.1.1 Site of recruitment

Previous experience at our site had taught us to expect an initial slow pace for screening and enrolment before gaining momentum, exhibiting an S-shaped enrolment rate curve.^29^ However, the initial pace was even slower than expected forcing a review of our recruitment methods. The initial recruitment plan was to approach mothers of infants attending public health clinics for the routine 6-week vaccination visit. This strategy failed due to limited space for study staff in the clinics and a reluctance of mothers to lengthen their clinic stay by engaging with our staff. A protocol amendment allowed us access to home addresses in the birth registers and vaccination records at local public health facilities which enabled our staff to visit parents at home resulting in substantially more effective recruitment.

#### 3.1.2 Staffing

Initial planning underestimated the number of staff needed to cope with the large numbers to be recruited and vast distances to be travelled. The study area is approximately 10,000 km^2^ and included the inhabitants of fifteen towns and hundreds of surrounding farms. We increased staff as follows: four additional field workers for recruitment (initially *n* = 28) and ten more research nurses (initially *n* = 6) for screening, vaccination, follow-up activities and safety assessments. In addition, we assigned staff to purely administrative duties and re-organised the study staff into teams with team leaders. This had a positive impact on recruitment.

#### 3.1.3 Informed consent

As professional nurses are a scarce resource needed for clinical tasks, we invited GCP-trained lay field workers to undergo training in the informed consent process. Competence was evaluated through role-play and successful candidates were accredited to conduct informed consent. This increased the pool of staff available to conduct informed consent which led to an increase in enrolment rates.

### 3.2 Screening procedures and application of inclusion/exclusion criteria

#### 3.2.1 Phlebotomy technique

Approximately 3% of parents/caregivers who consented to participate in the trial withdrew during the screening phase, for a variety of reasons. About 20% of those who gave a reason said they were distressed by the phlebotomy process. Study nurses received additional training in infant phlebotomy from a paediatrician, who emphasised the use of appropriate anatomical sites (cubital fossa, hand or foot before external jugular vein), swaddling and the use of sucrose solution as an analgesic.^30,31^ A limit of maximum three phlebotomy attempts was set and the need to explain the procedure to the mother or caregiver was emphasised.

#### 3.2.2 Haemolysis of blood specimens

Haemolysed safety blood samples could not be processed by the laboratory necessitating repeated phlebotomy. This was not always well received by participant's parents, placed an increased burden on the screening clinic numbers and had budgetary implications. We introduced centrifugation of all blood samples for biochemistry testing at 3750 rpm for 10 min at the trial site laboratory before transport and delivery of samples to the diagnostic laboratory 110 km away and this substantially reduced the rate of haemolysed samples.

#### 3.2.3 TB exposure

We found that staff in first contact with potential participants was over-interpreting the exclusion criterion related to household TB contact. A clear definition of the term “household TB contact” was developed by the study medical team which defined a household contact as follows: one who has been diagnosed with TB after the infant's birth, and spent more than 6 h daily in the same living space. If the household member has been diagnosed with pulmonary TB before the infant's birth, they would qualify as a household contact if he/she had not yet completed 2 months of TB treatment or was a treatment defaulter or had drug resistant TB. The decision to exclude an infant based on “household TB contact” was made only by an investigator.

#### 3.2.4 Thrombocytosis

Screening haematology tests revealed a high prevalence of thrombocytosis in otherwise healthy infants. More than 90% of infants screened had platelet counts above the local laboratory defined upper limit of normal (350 × 10^9^/L) with 51% of screening values greater than 500 × 10^9^/L. These reference ranges are derived from Western sources and not local data. Based on a haematologist's advice to interpret the results in a clinical context, we enrolled infants with thrombocytosis providing other haematological parameters were within the normal range and the infant was clinically well.

#### 3.2.5 Impact of routine immunisations

We found that the 14-week routine immunisation doses were often delayed, resulting in study exclusions when the 28 day window between the receipt of routine immunisations and administration of the study vaccine fell after the 154 day age cut-off for study vaccination. A protocol amendment was approved to reduce the window between routine vaccines and the investigational vaccine to 14 days and to increase the age of eligibility for vaccination to 26 weeks. This reduced exclusion of infants due to being out of the vaccination window.

A temporary global shortage of BCG threatened enrolment, as eligibility for participation required receipt of BCG within seven days of birth. The local Department of Health arranged for areas with excess stock to share this with the birthing units in our study drainage area and arranged for the first new batch of BCG stock to be distributed in our study area to minimise the impact of this shortage.

A national polio eradication campaign, targeting all children from birth to 5 years regardless of immunisation status also threatened to disrupt enrolment. The campaign involved the administration of two doses of trivalent oral polio, four weeks apart. An arrangement was reached with the local health authorities whereby administration of polio vaccine was delayed until after study vaccine administration.

### 3.3 Case accrual

#### 3.3.1 Efficacy follow up

Blinded case accrual was monitored throughout study follow up. About nine months prior to study closeout, it was determined that insufficient TB cases would be accrued by study end to ensure statistical significance for the efficacy objective. Twenty-one cases meeting the endpoint 1 definition had been accrued at that point, when 33–35 had been expected. An amendment to lengthen follow up by six months was submitted and approved to address this shortfall.

## 4 Discussion

Designing and operationalising this first efficacy trial of a TB vaccine candidate in infants was complicated by the lack of a single test to diagnose childhood TB. This necessitated a clear clinical endpoint definition, a lengthy follow-up period and a large sample size. In modern times, regulatory authorities and ethics committees, and investigators and sponsors are much more sensitised to participant rights particularly in trials conducted in vulnerable populations. We used the experience gained from the conduct of a large BCG trial^26^ and a neonatal cohort study of TB in^28^ infants to design and plan for this clinical trial. In practice, there was an evolutionary process with respect to study design and operational activities as the trial progressed.

### 4.1 Study design

A standard vaccine efficacy trial design has been described. An area warranting further discussion is the clinical endpoint selection. The diagnosis of TB in children is challenging as the disease is pauci-bacillary. Numerous scoring systems have been developed but no validated scoring system exists.^32^ Because there is no correlate of protection, TB vaccine efficacy trials depend on clinical disease endpoints.^33^ In a trial setting, it is imperative that endpoints are as specific as possible to ensure that efficacy measures are a true reflection of vaccine benefit. An attempt to determine an internationally agreed endpoint for TB vaccine efficacy trials in infants reached only a partial consensus.^24^ Using our experience from previous trials^23,26^ we developed a hierarchical, three tiered endpoint which we felt would be most suited to an infant TB vaccine efficacy trial (Table 1). While Graham et al.^34^ have published consensus endpoints for TB diagnostics studies, it was agreed that such endpoints may not be suitable for infant TB vaccine trials due to active case finding and a younger study population. Active case finding truncates further disease progression because of early isoniazid preventive therapy (IPT) and TB treatment; children younger than 2 years are at risk for rapid progression to military/meningitic TB in the absence of a classical respiratory symptomatic phase seen in older children. Our endpoints contain elements which are pathognomonic of TB – bacteriological confirmation by culture and/or nucleic acid amplification test (GeneXpert^®^, Cepheid) and clinical syndromes for TB meningitis, opthalmic TB and typical histological findings. However, these forms of TB are less common. A triad of features more commonly used to diagnose TB in children – evidence of latent TB infection, a chest X-ray compatible with TB and symptoms of TB – were added to the endpoint definition. All three elements were required to confirm a diagnosis of TB to ensure that the endpoint would be as specific as possible. Endpoint 2 allows a slightly less rigorous definition of TB disease than Endpoint 1, based upon the triad of TB exposure/infection, radiographic features and symptoms. Although the symptom and radiological criteria are identical, the Endpoint 2 criteria for TB exposure/infection are widened to include children with a lower TST threshold (10 mm) and children living in a household with a known smear positive sputum TB patient.

### 4.2 Operational activities

The study was more staff intensive than initially anticipated. Staff salaries comprise a large proportion of the costs of efficacy trials, but it would be false economy to reduce the staffing complement but then have to lengthen the trial to meet targets. On the other hand, we have shown that flexibility with regards to staff roles and responsibilities, changing the recruitment window, centrifugation of blood specimens prior to transport and good counselling with phlebotomy procedures are improvements that add minimally to trial costs but benefit recruitment progress.

The principal investigator is held accountable for informed consent. However, according to International Conference on Harmonisation (ICH) and South African GCP guidelines,^35,36^ he/she may delegate this to a suitably qualified person. Certain sponsors nevertheless require a medically qualified investigator to conduct consent. In developing countries, this is not always feasible, particularly when enrolling large numbers of participants. We have shown that non-medical staff when well trained with competency checks and frequent monitoring, were able to conduct informed consent which met international and local standards.

“Normal” ranges used by diagnostic laboratories around the world are often based on Western populations, as was the case with normal platelet ranges in our study. Other African research sites have experienced this problem as well.^37–41^ In clinical trials, this results in 1) the possible exclusion of healthy participants and 2) abnormal laboratory results being classified as adverse events in enrolled participants with subsequent repeat blood draws until ‘resolution’. Clinical trial data or specifically designed surveys of local healthy populations should be used to define normal ranges that are appropriate and relevant to the study setting and population for use in both patient care and clinical trials.

Stockouts of EPI vaccines do occur commonly in developing country settings and contingency plans need to be in place should trials require their prior administration as an inclusion criterion. National vaccination campaigns will affect trials where administration of other vaccines is an exclusion criterion. Unless solutions are found to these factors, a costly pause in enrolment may occur. Excellent communication channels between SATVI and the local health services at all levels served to minimise the impact of these potentially detrimental episodes during our enrolment phase.

## 5 Conclusion

We describe the first proof-of-concept trial of a new TB vaccine in infants since BCG. The rationale for specific efficacy endpoints is provided. We have described the challenges encountered by a site experienced in clinical trials with investigational medicinal products and the need for constant monitoring, evaluation and adaptation. The solutions we implemented will be of value in the planning of efficacy trials of other TB vaccine candidates.

## Figures and Tables

**Table 1:** Endpoint definitions.

TB case definition Endpoint #1
Any of the following numerical categories • Isolation of *M. tuberculosis* from any site. • Identification of *M. tuberculosis* by an approved molecular diagnostic technique from any site. • Histopathology diagnostic for tuberculosis disease (such as caseating granulomas) • Choroidal tubercle diagnosed by an ophthalmologist • Miliary pattern on chest X-ray in an HIV-negative infant • Clinical diagnosis of tuberculous meningitis (CSF protein >0.6 g/L and pleocytosis >50/mm^3^ with mononuclear cell >50%) with features of basal meningeal enhancement and hydrocephalus on head CT. • Vertebral spondylosis • A single smear/histology specimen positive for acid fast (or auramine positive) bacilli from a normally sterile body site. • One of the following: a) Two acid fast or auramine smears positive each from a separate collection morphologically consistent with mycobacteria from either sputum or gastric aspirate that are not found to be non-tuberculous mycobacteria bacteria on culture, OR b) QuantiFERON conversion from negative or indeterminate to positive, OR c) Tuberculin skin test ≥15 mm AND One of the following compatible radiographic features: a) Calcified Ghon focus, OR b) Pulmonary cavity, OR c) Hilar/mediastinal adenopathy, OR d) Pleural effusion, OR e) Airspace opacification, AND One of the following clinical manifestations: a) Cough without improvement for longer than two weeks, OR b) Weight loss of at least 10% of body weight for at least 2 months, OR c) Failure to thrive (crossing at least one entire major centile band downward) for at least 2 months, where the major centile bands are defined as <97th–90th, <90th–75th, <75th–50th, <50th–25th, <25th–10th, and <10th–3rd weight-for-age centiles.∗

TB case definition Endpoint #2
Any of the following numerical categories • Isolation of *M. tuberculosis* from any site. • Identification of *M. tuberculosis* by an approved molecular diagnostic technique from any site. • Histopathology diagnostic for tuberculosis disease (such as caseating granulomas) • Choroidal tubercle diagnosed by an ophthalmologist. • Miliary pattern on chest X ray in an HIV-negative infant • Clinical diagnosis of tuberculous meningitis (CSF protein >0.6 g/L and pleocytosis >50/mm^3^ with mononuclear cell >50%) or features of basal meningeal enhancement and hydrocephalus on head CT. • Vertebral spondylosis • A single smear/histology specimen positive for acid fast (or auramine positive) bacilli from a normally sterile body site. • One of the following: a) One acid fast or auramine smear positive morphologically consistent with mycobacteria from either sputum or gastric aspirate that are not found to be non-tuberculous mycobacteria bacteria on culture, OR b) QuantiFERON conversion from negative or indeterminate to positive, OR c) Residence in a household with an AFB smear positive member, OR d) Tuberculin skin test ≥10 mm AND One of the following compatible radiographic features: a) calcified Ghon focus, OR b) pulmonary cavity, OR c) hilar/mediastinal adenopathy, OR d) pleural effusion, OR e) airspace opacification, OR AND One of the following clinical manifestations: a) Cough without improvement for longer than two weeks, OR b) Weight loss of at least 10% of body weight for at least 2 months, OR c) Failure to thrive (crossing at least one entire major centile band downward) for at least 2 months, where the major centile bands are defined as <97th–90th, <90th–75th, <75th–50th, <50th–25th, <25th–10th, and <10th–3rd weight-for-age centiles.∗

TB case definition Endpoint #3
All individuals who are placed on anti-tuberculosis therapy with the intent of treating tuberculosis regardless of whether they have met the other efficacy endpoints

∗ Centers for Disease Control (CDC) Growth Charts (USA), developed by the National Center for Health Statistics in collaboration with the National Center for Chronic Disease Prevention and Health Promotion (2000).

**Table 2:** Study design and operational factors, implementation lessons learned, and alternative approaches and solutions.

Category	Design/Operational factor	Alternative/Solution
Recruitment	Site of recruitment	Ensure conducive environment for informed consent
	Sub-optimal field staff allocation	Ensure adequate staff allocation
	Informed consent	Train lay field workers in informed consent
Screening procedures and application of inclusion/exclusion criteria	Community attitudes to phlebotomy	Ensure correct paediatric phlebotomy technique and counsel parents about procedure.
	Specimen haemolysis	Centrifugation of specimens prior to transport
	Definition of TB exposure	Clear detailed definition – investigator assessment required
	Thrombocytosis – inappropriate laboratory reference ranges	Inclusion based on investigator clinical assessment
	Impact of routine immunisations	Widen vaccination window, be prepared for stockouts and mass immunisations campaigns
Case accrual	TB case accrual	Monitor case accrual and extend follow up if needed.
